# Effect of Gellan Gum on the Properties of Collagen-HPMC Freeze-Dried Hydrogels for Mucosal Administration

**DOI:** 10.3390/gels11100793

**Published:** 2025-10-02

**Authors:** Ioana Luca, Mădălina Georgiana Albu Kaya, Raluca Țuțuianu, Cristina Elena Dinu-Pîrvu, Maria Minodora Marin, Lăcrămioara Popa, Irina Titorencu, Valentina Anuța, Mihaela Violeta Ghica

**Affiliations:** 1 Department of Physical and Colloidal Chemistry, Faculty of Pharmacy, “Carol Davila” University of Medicine and Pharmacy, 6 Traian Vuia Str., 020956 Bucharest, Romania; ioana.luca@drd.umfcd.ro (I.L.); cristina.dinu@umfcd.ro (C.E.D.-P.); lacramioara.popa@umfcd.ro (L.P.); valentina.anuta@umfcd.ro (V.A.); mihaela.ghica@umfcd.ro (M.V.G.); 2 Innovative Therapeutic Structures Research and Development Center (InnoTher), “Carol Davila” University of Medicine and Pharmacy, 6 Traian Vuia Str., 020956 Bucharest, Romania; 3 Department of Collagen, Division of Leather and Footwear Research Institute, National Research and Development Institute for Textiles and Leather, 93 Ion Minulescu Str., 031215 Bucharest, Romania; 4 Cell and Tissue Engineering Department, “Nicolae Simionescu” Institute of Cellular Biology and Pathology, 8 B. P. Hasdeu Street, District 5, 050568 Bucharest, Romania; irina.titorencu@icbp.ro; 5 Advanced Polymer Materials Group, Faculty of Chemical Engineering and Biotechnology, National University of Science and Technology Politehnica Bucharest, 011061 Bucharest, Romania; maria_minodora.marin@upb.ro; 6 Research Institute for Advanced Materials, Products, and Processes (CAMPUS), National University of Science and Technology Politehnica Bucharest, 060042 Bucharest, Romania

**Keywords:** collagen, hydroxypropyl methylcellulose, gellan gum, freeze-dried hydrogels, wafers, mucosal administration

## Abstract

Mucosal drug delivery is gaining attention for its ability to provide localized treatment with reduced systemic side effects. The vaginal route has been proven effective for managing gynecological conditions, though it poses certain limitations. Biopolymers can help overcome these challenges by enhancing therapeutic efficiency and offering beneficial properties. This study aimed to develop and evaluate hydrogels and their freeze-dried forms (wafers) based on collagen, hydroxypropyl methylcellulose, and gellan gum. Initially, a collagen gel was obtained by extraction from calfskin, which was brought to a concentration of 1% and a physiological pH with 1 M sodium hydroxide solution. This gel was combined with either 2% hydroxypropyl methylcellulose gel, 1.2% gellan gum gel, or both, in different proportions. Thus, five mixed hydrogels were obtained, which, along with the three individual gels (controls), were lyophilized to obtain wafers. Furthermore, the hydrogels were assessed for rheological behavior, while the collagen structural integrity in the presence of the other biopolymers was evaluated using circular dichroism and FT-IR spectroscopy. The wafers were characterized for morphology, wettability, swelling capacity, enzymatic degradation resistance, and in vitro biocompatibility. All hydrogels exhibited non-Newtonian, pseudoplastic behavior and showed collagen structure preservation. The wafers’ characterization showed that gellan gum enhanced the hydrophilicity and enzymatic stability of the samples. In addition, the extracts from the tested samples maintained cell viability and did not affect actin cytoskeleton morphology, indicating a lack of cytotoxic effects. This study emphasizes the importance of evaluating both the physicochemical properties and biocompatibility of biopolymeric supports as a key preliminary step in the development of vaginal drug delivery platforms with biomedical applications in the management of gynecological conditions.

## 1. Introduction

In recent years, mucosal drug delivery has gained significant attention as an alternative to oral and parenteral conventional routes due to its non-invasiveness, ease of administration, and the possibility of developing targeted therapies, while minimizing the systemic side effects [1]. Mucosal drug delivery includes ophthalmic, nasal, oral, rectal, and vaginal routes, each offering either direct access to systemic circulation or localized treatment with reduced first-pass metabolism effects [2]. Among these, vaginal drug delivery has shown great promise for locally managing several gynecological conditions [3]. It is commonly employed for treating infections of different etiologies [4,5], inflammation [6], or vaginal atrophy [7] and has shown promise in the local treatment of cervical intraepithelial neoplasia [8]. The advantages of vaginal delivery include localized drug administration, sustained release potential, and avoidance of gastrointestinal degradation of the drug, which is frequently encountered in oral administration [3,9]. However, it also presents some important drawbacks, one of the most prominent being represented by the potential for leakage, and therefore, low residence time at the vaginal level [3,9,10].

Polymers used in topical and mucosal drug delivery offer several advantages that help address the challenges associated with these routes of administration. Many natural and synthetic polymers are commonly used due to their biocompatibility, mucoadhesive properties, and ability to control drug release [11,12]. Mucoadhesive polymers can prolong the residence time of drugs at the site of action, enhancing their bioavailability and overall treatment efficiency [13]. These characteristics make polymers ideal candidates for overcoming issues such as rapid clearance of the drug and local irritation [14,15,16]. Moreover, natural polymers are safe and biocompatible choices for the development of drug delivery systems [17].

Collagen is the key structural protein in the extracellular matrix, known for its excellent biocompatibility, low immunogenicity, and ability to support cell adhesion, proliferation, and tissue regeneration [18]. Due to these properties, collagen is extensively used in various biomedical applications, including wound dressings [19,20], tissue engineering [21], drug delivery systems [22,23,24], and cosmetics [25]. Along with elastin, collagen plays an essential role in maintaining the structural integrity and elasticity of the vaginal walls, with types I and III being the most predominant. When collagen levels decline, whether due to aging or hormonal changes, the vaginal mucosa becomes thinner, less elastic, and more prone to damage. Moreover, stimulating collagen production through heat-based therapies has been shown to enhance tissue firmness, promote vascular regeneration, and restore overall vaginal health [26]. Collagen-based formulations have been shown to support tissue repair and stimulate the regeneration of the mucosal epithelium, making it a promising therapeutic option for conditions such as vaginal atrophy, cervicovaginal inflammation, and infections, promoting re-epithelialization in inflammatory and dystrophic conditions affecting the vaginal mucosa [6,27,28].

However, weak mechanical properties of collagen scaffolds [29], along with challenges related to gravitational forces and the dynamic environment of the vaginal canal, can limit their practical applications [9]. Factors such as the vaginal anatomy, biological fluids, and changes in body position or movement influence the residence time of the formulation and effective contact with the mucous membrane [9,30]. To address these limitations, collagen scaffolds can be either crosslinked using chemical or physical techniques, or enhanced through the addition of natural, semi-synthetic, or synthetic polymers [29,31]. In this regard, hydroxypropyl methylcellulose (HPMC), a well-known mucoadhesive cellulose derivative, could improve some of the properties of collagen-based polymeric systems and also facilitate controlled drug release and a prolonged residence time on the mucosal surfaces [32,33]. Moreover, the combination of collagen and HPMC leads to biocompatible polymeric systems with improved structural integrity and mucoadhesive capacity, making them particularly suitable for mucosal drug delivery [34,35]. In the initial stage of our research, we evaluated the influence of two mucoadhesive polymers—HPMC and carbomer—on the structure and properties of collagen. The results showed that the addition of HPMC led to an improvement in certain characteristics of collagen-based formulations. Furthermore, collagen-HPMC-based polymeric systems were found to be biocompatible, promoting fibroblast proliferation and migration, and, therefore, demonstrated potential for mucosal drug delivery applications [36].

Therefore, this study focused on improving the previously developed polymeric systems by incorporating a new biopolymer with special characteristics and evaluating its influence on collagen-HPMC-based biomaterials. Gellan gum (GG) is a microbial water-soluble exopolysaccharide produced by Sphingomonas species, initially discovered in 1978. It has a linear structure, composed mainly of β-D-glucose, β-D-glucuronate, and α-L-rhamnose, and a polyanionic character that makes it responsive to cationic environments. In a biomedical context, its ability to form gels when placed in contact with biological fluids that are rich in cations, such as Na^+^, Ca^2+^, or Mg^2+^, makes it a promising candidate for targeted drug delivery systems. Its ion-sensitivity facilitates in situ gelation at mucosal sites, including the vaginal mucosa [37]. It also possesses some excellent properties, such as resistance to temperature, stability in acidic environments, high swelling capacity, mucoadhesion, biodegradability, and biocompatibility, which make it a great candidate for drug delivery via various mucosal routes [38,39,40,41].

Several studies have highlighted the potential of gellan gum in vaginal drug delivery systems, owing to its ion-responsiveness, mucoadhesive properties, and biocompatibility. Gellan gum has been used to create in situ gelling formulations for the vaginal delivery of antibiotics such as clindamycin [42] or metronidazole [43], facilitating sustained drug release, improved mucoadhesion, and a lack of irritating effects. Thiolated gellan gum has been used in the development of vaginal films with enhanced adhesion and effective antimicrobial delivery [44]. Additionally, hybrid systems combining gellan gum with other polymers, such as HPMC [42] or poloxamers [43], have improved gel stability and retention. Nanocellulose-gellan gum scaffolds have shown promise for the delivery of an antifungal drug, ensuring prolonged release and efficient mucosal permeation [45]. All these studies highlight gellan gum’s versatility and effectiveness in the development of some safe and bioadhesive vaginal drug delivery systems. In addition to its widespread use in drug delivery through various mucosal routes, such as ocular [46,47], nasal [48,49], buccal [50,51], or vaginal [52], among others, gellan gum has demonstrated great potential in various biomedical applications, such as tissue engineering [53] and wound healing [54]. Its ability to form biocompatible, injectable hydrogels makes it a good candidate for cell encapsulation, providing a supportive three-dimensional matrix that promotes cell proliferation and function [54]. For example, gellan gum-based hydrogels have been used to encapsulate chondrocytes [55] and stem cells for cartilage regeneration [56]. Furthermore, gellan gum scaffolds have supported regeneration in other tissues, such as intervertebral discs [57,58] and skeletal muscle [59], where functionalized hydrogels have enhanced cell growth, vascularization, and tissue remodeling. Moreover, an injectable gellan gum-based hydrogel promoting osteogenesis and angiogenesis has shown improved bone regeneration and neovascularization in an animal model [60]. Additionally, gellan gum’s film-forming and moisture-retaining properties contribute to wound healing, creating a protective barrier that supports tissue repair, and can be loaded with antimicrobial or growth factors to accelerate healing [61,62]. Consequently, gellan gum’s properties make it an important biomaterial suited not only for controlled drug release, but also for regenerative medicine and wound healing applications.

Therefore, in the present study, collagen was combined with HPMC and gellan gum with the aim of improving its mechanical properties and potentially enhancing the formulation’s retention at the site of action, which is particularly relevant for vaginal application. To the best of our knowledge, no other studies have attempted to develop vaginal drug delivery supports based on collagen, HPMC, and gellan gum. Furthermore, no other studies evaluated the influence of gellan gum on collagen and HPMC-based biomaterials. Therefore, the aim of this study was to evaluate the influence of gellan gum on collagen and HPMC-based biopolymeric systems and to further characterize the obtained formulations, with possible future application as vaginal drug delivery carriers.

## 2. Results and Discussion

**Figure 1:**
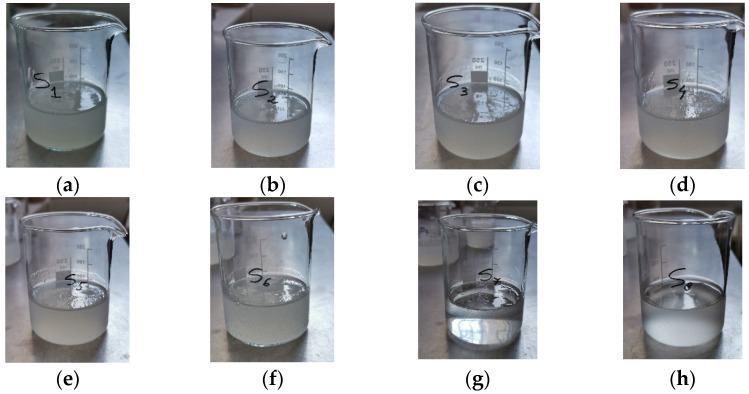
Hydrogels based on collagen, HPMC, and GG: (**a**) sample S1, (**b**) sample S2, (**c**) sample S3, (**d**) sample S4, (**e**) sample S5, (**f**) sample S6, (**g**) sample S7, and (**h**) sample S8.

Various hydrogel formulations were prepared using three individual hydrogels, namely 1% collagen gel, 2% HPMC gel, and 1.2% GG gel, mixed in different proportions. These hydrogels were subsequently lyophilized to obtain wafers. The hydrogels and their freeze-dried forms (wafers) were then analyzed by physicochemical characterization methods and biocompatibility evaluation. The eight obtained hydrogels (S1–S8) can be observed in Figure 1.

Except for the 100% HPMC hydrogel (S7), which was distinguished by clarity and transparency, the other hydrogels had an opalescent, white-yellowish appearance of variable intensity due to the proportion of collagen and/or gellan gum hydrogels present in their composition.

**Figure 2:**
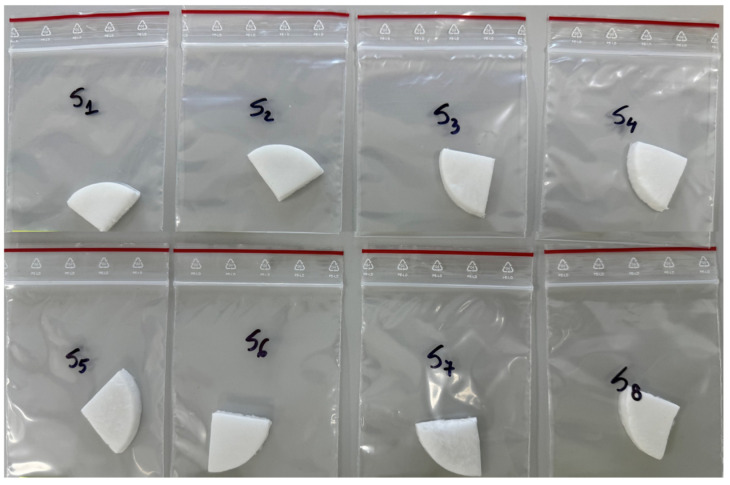
Freeze-dried hydrogels (wafers) based on collagen, HPMC, and GG (S1–S8).

The freeze-dried hydrogels (wafers), obtained through the lyophilization of the eight hydrogels and then cut into smaller pieces for further analysis, are presented in Figure 2.

### 2.1. Evaluation of the Rheological Behavior of Designed Hydrogels

Upon contact with vaginal fluid, freeze-dried hyrogels absorb moisture and undergo a structural transformation into the corresponding hydrogel. This transition, induced by hydration, allows the sponge in dry form to swell and adhere to the vaginal mucosa, improving retention and surface contact. As the polymeric matrix becomes hydrated, it forms a soft, bioadhesive hydrogel that enables targeted and sustained release of the active ingredients. Therefore, this gradual transformation can lead to reduced leakage of the formulation and improved patient comfort [63,64,65].

The rheological behavior is an important attribute in the development of vaginal formulations because it directly influences key performance parameters such as spreading and retention on mucosal surfaces. Rheological properties dictate how well a gel can coat the vaginal epithelium without rapid leakage, having an adequate residence time and proper efficiency. Moreover, the optimal rheological behavior can enhance patient comfort and compliance [66,67].

**Figure 3:**
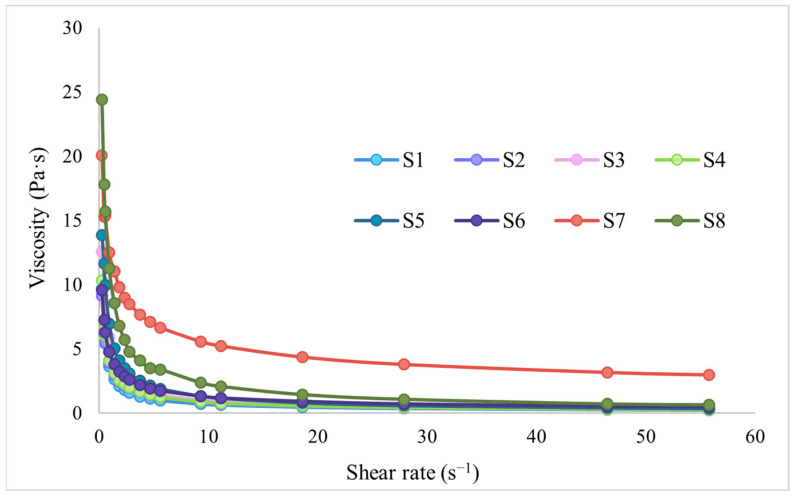
Viscosity versus shear rate rheograms for collagen, HPMC, and GG hydrogels.

First, to evaluate the rheological behavior of the designed hydrogels, the rheograms illustrating viscosity as a function of shear rate were represented (Figure 3).

Analyzing the rheograms in Figure 3, a decrease in the viscosity of all hydrogels based on collagen, HPMC, and GG is highlighted, simultaneously with the increase in the shear rate, thus confirming their pseudoplastic, non-Newtonian character, under the test conditions. This type of behaviour of hydrogels is a desirable feature in the development of semisolid systems for vaginal drug delivery, which favors both easy administration and appropriate local distribution at the site of action [67].

To further quantify the flow behavior of the designed hydrogels, the Power law model [68] was used, which describes the relationship between viscosity and shear rate:

(1) $$\eta =m\cdot {\dot{\gamma }}^{-n}$$

where m (consistency index, also found as “k”) represents the viscosity corresponding to a shear rate of 1 s^−1^, and n is the flow index [69].

**Figure 4:**
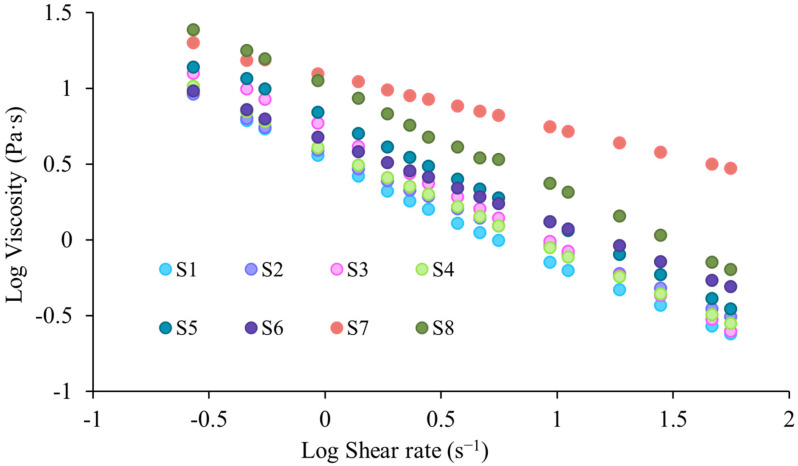
Log-log viscosity versus shear rate rheograms for collagen, HPMC, and GG hydrogels.

The log-linearization of Equation (1) led to obtaining the specific descriptors of the Power law model [68]. The log-log viscosity versus shear rate rheograms obtained for collagen, HPMC, and GG-based hydrogels are shown in Figure 4. The values obtained for the parameters m, n, and the coefficient of determination R^2^ are shown in Table 1.

Thus, from the obtained results, it can be seen that for all tested hydrogels, values of the coefficient of determination R^2^ higher than 0.9900 were recorded, confirming that the experimental data accordingly verify the Power law model. The pseudoplastic, non-Newtonian character (shear thinning) can also be confirmed by analyzing the values obtained for the aforementioned n parameter, as a descriptor of the Power law model, also called the flow index. Depending on the n value, pharmaceutical systems can have either a Newtonian (n = 1) or non-Newtonian behaviour when n ≠ 1 (pseudoplastic flow—n < 1, dilatant flow—n > 1) [68,69]. Analyzing the values obtained in the present study for the n parameter, it can be seen that it had values of <1 for all of the analyzed hydrogels. The most pronounced pseudoplastic character was highlighted in the case of sample S7 (HPMC 100%), which presented the lowest flow index value (n = 0.35), followed by sample S6 (50% collagen, 25% HPMC, 25% GG) with a flow index of n = 0.56. With the exception of the two previously mentioned hydrogels (S6, S7), all other hydrogels showed close values of the flow index, between 0.62–0.75, also being less than unity. The S8 sample corresponding to the gellan gum hydrogel showed a similar n value to that obtained for the collagen gel (S1), with these samples displaying a similar rheological behaviour, which is possibly linked to their natural origin and could imply a more heterogeneous structure. The highest flow index, corresponding to a weaker pseudoplastic character, was found in sample S3 based on 75% collagen and 25% GG, followed by sample S5 (50% collagen, 50% GG) and by sample S8 (100% GG). Thus, it can be stated that gellan gum, having a weaker pseudoplastic character, does not lead to an improvement in the flow properties of the collagen gel. At the same time, evaluating the samples that also had HPMC in their composition (S2, S4, S6), it can be seen that the cellulose derivative manages to favor a more pronounced pseudoplastic character for these samples. Regarding the consistency index, m, samples S7 (100% HPMC gel) and S8 (100% GG gel) showed significantly higher values compared to collagen-based sample S1, which displayed the smallest m value. Furthermore, the addition of 25% gellan gum gel to the collagen gel (S3) led to a greater increase in the consistency index compared to the addition of the same amount of HPMC gel (S2). Sample S4, based on 75% collagen gel and equal proportions (12.5%) of HPMC and gellan gum gels, had an intermediate consistency index between that of sample S2 (75% collagen and 25% HPMC) and that of sample S3 (75% collagen and 25% gellan gum). Furthermore, comparing samples S5 and S6, with 50% collagen gel, the sample to which only gellan gum gel was added (S5—50% collagen gel, 50% GG gel) had a greater increase in the consistency index compared to the sample to which both GG and HPMC gels were added (S6—50% collagen gel, 25% GG gel, 25% HPMC gel). Thus, we can conclude that gellan gum had a greater influence on the consistency of the combined hydrogels, while HPMC had a greater influence on their pseudoplasticity. Achieving a balance in terms of these rheological properties is important, with pseudoplasticity ensuring easy administration and adequate mucosal coating, while the consistency index can be an indicator of the cohesion of the polymeric system, which is important for ensuring an adequate mucosal retention time, under conditions of low shear [67,70].

### 2.2. Circular Dichroism (CD)

**Figure 5:**
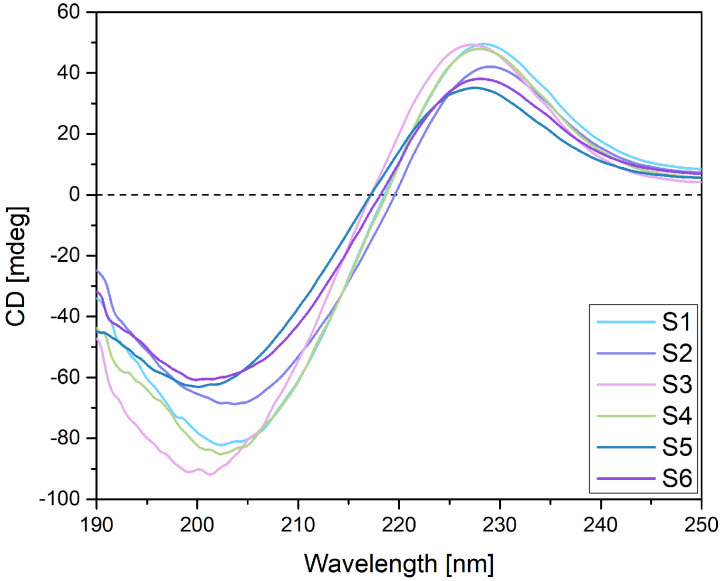
CD spectra obtained for collagen-based hydrogels.

Circular dichroism analysis was performed in order to assess the influence of HPMC and GG on the triple-helical conformation of collagen. The preservation of collagen’s triple-helical structure is critical to its biological function, as this conformation provides the mechanical stability, flexibility, and proteolytic resistance necessary for maintaining connective tissue integrity, facilitating wound healing and bone repair [71,72,73,74]. Unlike other extracellular matrix (ECM) proteins, collagens are defined by this triple-helical conformation, which also plays a central role in regulating physiological processes such as endothelial cell migration and vascular repair. Several sequences within the triple-helical domains of collagens have been identified as key sites for cell binding, emphasizing that the integrity of the triple helix is essential not just for maintaining mechanical function but also for preserving conformation-dependent cell signaling and adhesion [74,75]. The CD spectra obtained for the combined collagen-based hydrogels (S2–S6) were similar to those obtained for the collagen gel (sample S1), in which a positive absorption band with a maximum at 228.42 nm, a negative absorption band with a minimum at 202.40 nm, and a zero ellipticity point located at 218.57 nm were highlighted. These results are consistent with those identified in the specialized literature and in our previous study [36,76,77,78]. In addition, the CD spectrum obtained for sample S1 (100% collagen gel) correlates with the information found in the literature regarding the spectrum of native collagen, which is characterized by a positive peak located between 210 and 230 nm and a negative peak that can be visualized in the 190–204 nm region [79,80]. This shows that the triple-helical structure of collagen was most likely preserved in the gel. For the hydrogels corresponding to samples S2–S6, both the positive and negative absorption bands were revealed, with minima and maxima located at wavelengths close to those identified in the CD spectrum of the collagen gel (S1). Moreover, by analyzing the obtained spectra and considering that the disappearance of the positive absorption band has been linked to collagen denaturation [78], it can be concluded that the addition of HPMC and gellan gum did not disrupt collagen structure. Also, the zero ellipticity point was similar for the analyzed hydrogels, located between 217.20 and 219.60 nm. The CD spectra obtained for the hydrogels based on collagen and natural mucoadhesive polymers (HPMC and gellan gum) are shown in Figure 5.

Furthermore, by analyzing the differences noticed in the obtained CD spectra, compared to that of the collagen gel (sample S1), a decrease in both positive and negative ellipticity could be observed, which was more pronounced in the case of S5 and S6 samples with 50% collagen gel in their composition. This decrease could be correlated with a weaker CD signal given by the reduced proportion of collagen in these samples. The different proportions of the other biopolymers in these samples (50% GG gel—sample S5 vs. 25% HPMC gel, 25% GG gel—sample S6) did not cause significant differences between the spectra of the two samples. Sample S2 (75% collagen gel, 25% HPMC gel) also showed a decrease in ellipticity compared to the CD spectrum of the collagen gel, but this was less pronounced compared to that observed in the S5 and S6 samples, which may highlight some influence of the interactions between collagen and HPMC on collagen structure. This can also be highlighted by comparing the samples with a collagen gel content of 75% (S2, S3, S4), of which sample S2 had the most significant decrease in ellipticity compared to the spectrum of the S1 sample. Samples S3 and S4, with a percentage composition of collagen gel of 75% and either one of the other two biopolymers (S3—GG 25%) or both (S4—HPMC 12.5%, GG 12.5%), presented spectra that were very similar to that of sample S1 (100% collagen gel), confirming the preservation of the structural integrity of the collagen in these samples. Moreover, an increase in negative ellipticity could be observed for sample S3, which was based on 75% collagen gel and 25% GG gel, compared to S1, which could be indicative of a certain stabilizing effect of GG towards the collagen triple-helical structure.

### 2.3. Fourier Transform Infrared (FT-IR) Spectroscopy

The FT-IR spectrum obtained for sample S1 (collagen 100%), showed the presence of absorption bands specific to collagen: amide A—3275 cm^−1^, which can be attributed to the stretching vibration of the N-H bond; amide B—2955 cm^−1^, associated with the asymmetric stretching of the CH_2_ group; amide I—1635 cm^−1^, which can be correlated with the stretching vibration of the carbonyl bonds in the polypeptide skeleton, coupled with the stretching vibration of the CN bond; amide II—1541 cm^−1^ and amide III—1230 cm^−1^, these peaks remained visible in all samples containing collagen. The presence of the specific Amide I absorption band in the obtained spectra is considered an indicator of collagen secondary structure integrity [81,82]. Also, the spectra recorded for the combined freeze-dried hydrogels showed the specific peaks of the two mucoadhesive biopolymers, namely HPMC and GG. Regarding the FT-IR spectrum obtained for the S7 sample (HPMC 100%), a series of specific peaks located at wavelengths close to those identified in the specialized literature, namely at 3377 cm^−1^, correlated with the stretching vibration of the hydroxyl groups, at 2867 cm^−1^—possibly due to the stretching vibration of the C-H groups and at 1059 cm^−1^—possibly correlated with the stretching vibration of the C-O bond [35,83]. The FT-IR spectrum obtained for the S8 wafer (GG 100%), contains a series of characteristic peaks that can be identified at 3339 cm^−1^, possibly due to the stretching vibration of the hydroxyl groups, 2873 cm^−1^—attributed to the asymmetric stretching of the CH_2_ group, 1614 cm^−1^ and 1385 cm^−1^—possibly related to the stretching vibrations of the carboxyl groups and at 1030 cm^−1^, attributed to the stretching of the C-O bond [84,85]. The FT-IR spectra obtained for the samples based on collagen-HPMC, collagen-GG, or those containing all three biopolymers, revealed the presence of the absorption bands that are characteristic of collagen, as well as those specific to HPMC and gellan gum, but with changes in their position or intensity that can be attributed to the interactions established between the three biopolymers. According to literature, a shift of the amide I band toward lower wavenumbers is typically associated with a decrease in molecular order [82]. In the present study, the amide I band position in the spectra of the combined samples remains quasi-stationary in most cases, suggesting that the molecular order and thus, the integrity of the triple-helical structure, is largely preserved. However, exceptions were observed for samples S5 and S6, both of which contain 50% collagen. In these cases, the amide I band shifted from 1635 cm^−1^ (as observed in the pure collagen gel, sample S1) to 1620 cm^−1^ and 1624 cm^−1^, respectively, indicating a potential reduction in molecular order. These results could be in agreement with those obtained in circular dichroism analysis, where these samples recorded a more pronounced decrease in both positive and negative ellipticity, compared to the other samples. Regarding both HPMC and GG, a peak around 1000 cm^−1^ can be observed, which is, as stated before, likely associated with the stretching vibration of the C–O bond. From the obtained spectrum of sample S1, it can be observed that this peak is absent in the collagen spectrum. Notably, the intensity of this band increases in the composite samples, reaching its maximum in formulations containing all three polymers. This enhancement may result from the overlapping contributions of the C–O stretching vibrations from both HPMC and GG. The highest intensity is observed in sample S6, based on a 50% collagen gel and equal proportions of HPMC and GG gels (25% each), suggesting a cumulative effect of the two biopolymers. Additionally, for sample S6, the peak observed in the 3500 cm^−1^ region—associated with hydroxyl groups—also shows increased intensity, possibly due to the inclusion of the two hydroxyl-rich polymers (HPMC and GG), which could significantly contribute to that specific region of the spectrum. This could also suggest the formation of new hydrogen bonds in this sample and the presence of a greater amount of bound water due to the water affinity of the two polymers.

**Figure 6:**
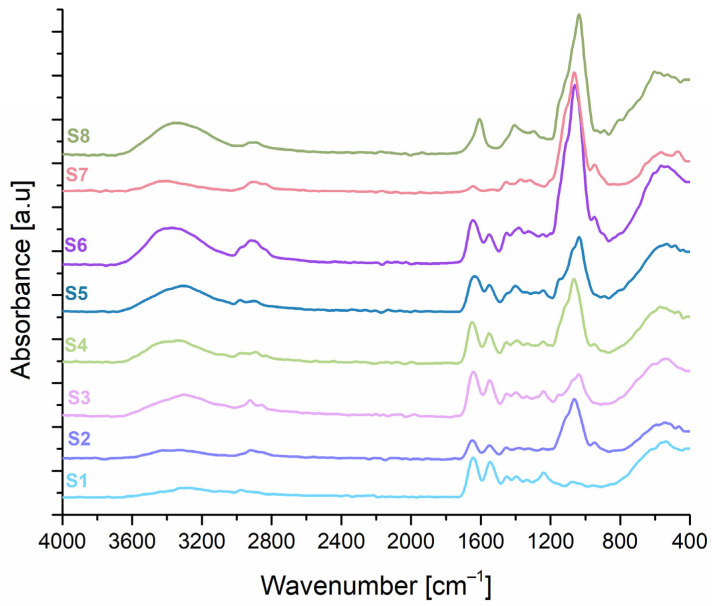
FT-IR spectra obtained for collagen-, HPMC-, and GG-based wafers.

The FT-IR spectra obtained for collagen, HPMC, and GG freeze-dried hydrogels are shown in Figure 6.

### 2.4. Scanning Electron Microscopy (SEM)

**Figure 7:**
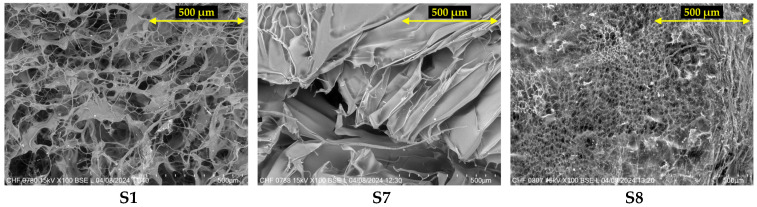
SEM images obtained for control samples S1, S7, and S8.

**Figure 8:**
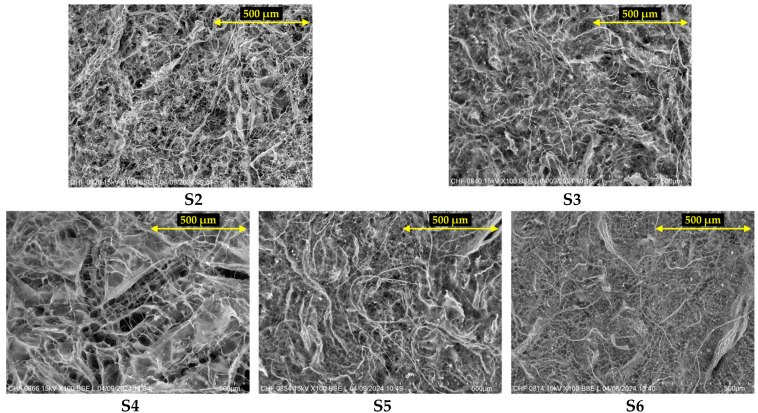
SEM images obtained for samples S2–S6 representing the combined wafers (100× magnification).

The evaluation of S1–S8 wafers by scanning electron microscopy allowed the identification of the characteristic morphology of each polymer in the composition of the samples. The images obtained for the control samples (S1—collagen 100%, S7—HPMC 100%, and S8—GG 100%) are presented in Figure 7, while the microstructure of the combined matrices (S2–S6) is shown in Figure 8.

Collagen is known for its microporous structure, with pores of different sizes, interconnected by networks of fibers and fibrils [86,87], which can be observed in Figure 7. On the other hand, sample S7, based on 100% HPMC, showed a more compact sheet-like structure. In this regard, based on literature findings, hydrogels with more compact microstructures tend to have lower water absorption capacity [88], and therefore, it is expected that the S7 sample, exhibiting a denser and more compact structure, will demonstrate a reduced swelling capacity. As for gellan gum, it has an intermediate microstructure between the porous one, with a polymer network resembling a spiderweb, and the compact, dense one is similar to cellulose derivatives [88,89].

Furthermore, depending on the proportions of polymers found in each sample, the specific morphology could be observed. Sample S2, based on collagen and HPMC, had a denser structure compared to S1, with smaller pores and a more compact fibril entanglement. Sample S3, based on collagen and GG, combines the features of both polymers with a layered microporous appearance and is very similar to the image obtained for the S5 sample, also based on collagen and GG, but with a higher proportion of GG. The image obtained for S4 sample based on all three polymers, has a distinctive appearance, which seems to be most influenced by the structure of collagen and HPMC, featuring large pores embedded in a more compact, multilayered structure. Comparatively, sample S6, which also contains all three polymers, but with a higher proportion of the two mucoadhesive polymers—HPMC and GG (25% each)—appears to have a structure that is very similar to that of gellan gum, featuring numerous small pores with fibrous regions distributed among them.

### 2.5. Surface Wettability of Freeze-Dried Hydrogels

**Figure 9:**
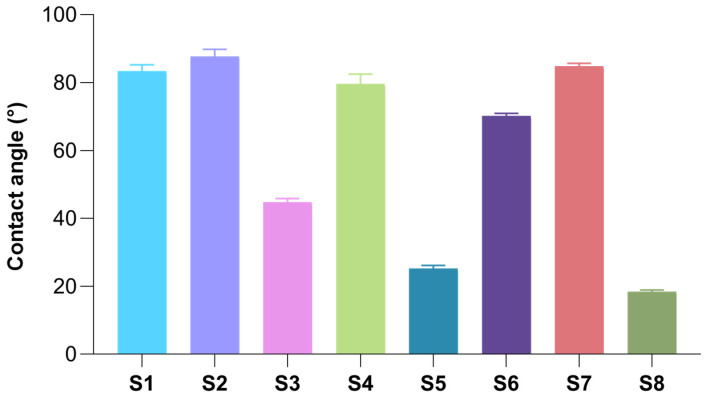
Comparative illustration of the average contact angle values recorder for S1–S8 samples.

Hydration plays a pivotal role in mucoadhesion at mucosal surfaces, as it facilitates polymer chain relaxation and interpenetration with mucin [90]; therefore, assessing the surface wettability of vaginally intended wafers is essential to ensure adequate hydration within the vaginal canal for effective and sustained adhesion. However, maintaining a proper balance in terms of hydration also requires careful control of swelling since excessive swelling can compromise adhesion duration despite sufficient polymer–mucin interactions [90]. For assessing the surface wettability, the contact angle method was used. In the case of wafers, the contact angle is rather dynamic and not static due to the porosity of these biomaterials [91]. Nevertheless, following preliminary tests, an increased hydrophilicity was observed, especially for gellan gum-based wafers, which did not allow the recording of gradually decreasing contact angle values. Taking this into account, for the present analysis, only the first contact angle value was recorded, corresponding to the initial contact between the droplet of SVF and the wafer surface. The experiment was performed in triplicate, and the reported values represent the average of three determinations. The comparative illustration of the results obtained within the contact angle measurements are presented in Figure 9.

For the S1 sample (collagen 100%), an average contact angle value of 83.34° < 90° was obtained, suggesting a weak hydrophilic nature of the sample. Regarding the S7 sample (HPMC 100%), a behaviour similar to that of the collagen wafer was observed, recording an average contact angle value of 84.85°. In addition, the S2 sample, based on 75% collagen and 25% HPMC, also had a similar behavior to matrices S1 and S7; in this case, a contact angle of 87.70° was identified, possibly due to the denser polymeric network of this sample. At the opposite pole, the average value of the contact angle recorded for the S8 sample (GG 100%) was 18.38°, suggesting a strong hydrophilic character. This hydrophilic character was evident for all samples that had gellan gum in their composition, more or less, depending on the proportion of gellan gum in the sample. Sample S3 (75% collagen, 25% GG) had an intermediate behavior between samples S1 and S8, presenting a hydrophilic character and an average value of the contact angle of 44.71°. Regarding matrices S4 and S6, containing all three studied polymers, similar average contact angle values of 79.55° and 70.18°, respectively, were revealed, with a lower value obtained for sample S6 with a higher percentage of gellan gum. These values could be justified by the majority presence of collagen and HPMC in the samples, which previously showed a weak hydrophilic character, together with gellan gum in a lower proportion, which improved the hydrophilicity of the samples, resulting in lower average contact angle values, compared to those obtained for matrices S1, S2, and S7. Regarding the S5 sample, with an equal content of collagen and gellan gum, an average value of the contact angle of 25.20° was recorded, indicating a high hydrophilicity of this sample, justified by the high content of gellan gum (50%).

### 2.6. Swelling Capacity of Freeze-Dried Hydrogels

The low amount of fluid present intravaginally and the constant production and elimination of cervicovaginal mucus, which acts as a protective barrier, can negatively impact vaginal drug delivery systems [92,93,94]. Therefore, wafers designed for vaginal drug administration should rapidly absorb the limited available moisture, adhere effectively to the mucosal surface, and resist being eliminated from the vaginal canal [64]. Moreover, swelling capacity plays a key role in modulating drug release, as it influences how fluids enter the matrix and how the drug dissolves and diffuses from the swollen matrix [95].

**Figure 10:**
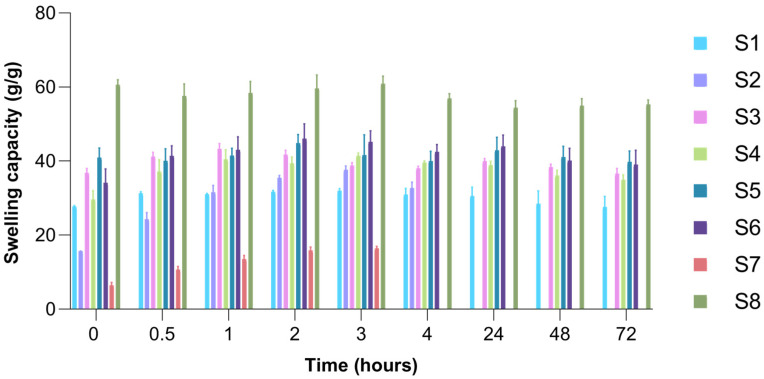
The swelling capacity (g/g) of the freeze-dried hydrogels at different time intervals.

The collagen-based wafer (S1) reached a maximum of 32.567 g/g at 1.5 h after immersion in the simulated vaginal fluid and had a quasi-constant swelling capacity throughout the 72 h of the experiment, maintaining its structural integrity until the end point. As expected, the S7 sample based on 100% HPMC had a lower swelling capacity, which can be correlated with SEM images, reaching a maximum of 16.37 g/g at 3 h after immersion in simulated vaginal fluid, then losing its structural integrity, which can be correlated with the solubility in water of this polymer [96]. The freeze-dried hydrogel based on 100% gellan gum (S8) presented the highest swelling capacity among the analyzed samples, reaching a maximum of 60.93 g/g at 3 h after immersion in the fluid and maintaining its structural integrity throughout the experiment. The comparative illustration of the swelling capacity of S1–S8 wafers at different time intervals is shown in Figure 10.

The combined wafers showed an intermediate behaviour, depending on the polymers in their composition and their proportions. The S2 sample had a maximum swelling capacity of 37.64 g/g, which was reached 3 h after the start of the experiment, but lost its structural integrity after 24 h. The samples based on collagen and gellan gum showed good swelling capacity, superior to those based on collagen or collagen-HPMC, and maintained their structural integrity throughout the experiment. As for the samples that contained all three polymers, they also maintained their structural integrity throughout the 72 h and showed a maximum swelling capacity of 41.420 g/g (S4 sample) and 46.122 g/g (S6 sample), respectively, recorded 3 and 4 h after the beginning of the experiment.

Among the polymers used, both collagen and gellan gum were responsible for maintaining structural integrity, with gellan gum also displaying superior hydrophilicity, reflected in the high absorption capacity of the simulated vaginal fluid recorded for samples with a high content of this polymer. The high fluid uptake capacity of the matrices that contained gellan gum was confirmed by data found in the literature [97], and its hydrophilicity was also highlighted in our surface wettability studies. All mixed samples showed intermediate swelling capacity between collagen (S1) and gellan gum (S8) wafers, which aligns with the preferred characteristics of the dosage forms intended for vaginal administration to support patient comfort [98].

### 2.7. Enzymatic Degradation of Freeze-Dried Hydrogels

In order to estimate the in vivo stability of collagen wafers, their degradation behavior under in vitro enzymatic conditions can be analyzed, as collagenases—whether extracellular or intracellular—are the only enzymes capable of fully degrading collagen [99]. Given that vaginal isolates of *Prevotella bivia*, *Prevotella disiens*, *Sneathia amnii*, and *Porphyromonas* spp. have been found to produce collagenases in the context of bacterial vaginosis-associated dysbiosis or pre-term birth [100,101,102], we conducted in vitro enzymatic degradation tests using collagenase solution to simulate potential biomaterial degradation in such an environment.

**Figure 11:**
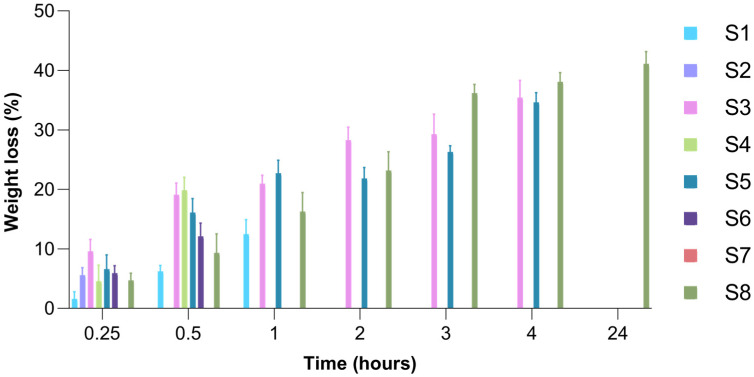
Weight loss (%) recorded for S1–S8 freeze-dried hydrogels over 24 h.

The 100% collagen-based wafer (S1) showed a weight loss of 14.30% recorded at 1 h after the addition of collagenase solution, followed by partial degradation with the loss of structural integrity and complete transformation into the original collagen gel after 24 h. The results obtained for S7 sample (HPMC 100%) showed that its transformation into the corresponding hydrogel took place in the soaking step, prior to the addition of the collagenase solution, which correlates well with the swelling capacity results and could be due to the water solubility of HPMC. As expected, the wafer based on 100% gellan gum (S8) was not influenced by collagenase activity and recorded a maximum weight loss of 69.91% at 24 h after the addition of collagenase solution. The loss of its structural integrity and its transformation into the original hydrogel occurred gradually, between 24 and 48 h from the start of the experiment. However, if we consider the results obtained in the swelling capacity analysis, it is possible that the collagenase solution had some influence on its degradation rate, transforming more quickly into the corresponding hydrogel. The results obtained in the assessment of the degree of enzymatic degradation suffered by S1–S8 wafers are shown in Figure 11. The S2 sample (collagen/HPMC ratio—3:1) suffered a minimal weight loss of 3.47% in the first 15 min of the experiment, rapidly transforming into the original hydrogel. The rapid degradation of this sample can also be attributed to the water solubility of the cellulose derivative from its composition, correlating with the data obtained by evaluating the swelling capacity of the wafers. The S3 and S5 matrices that were based on collagen and gellan gum maintained their structural integrity until they were close to reaching the 24 h threshold, registering a maximum weight loss of 52.62% and 53.06%, respectively, at 4 h after contact with the collagenase solution. The S4 and S6 wafers, which had all three polymers in their composition, degraded under the action of the collagenase solution in the first hour of the experiment, recording a final quantifiable weight loss of 23.78% and 13.83%, respectively, 30 min after contact with the collagenase solution. From the results obtained, it can be seen that the presence of gellan gum in the composition of the samples, along with collagen, led to a delay in their degradation. Thus, the only collagen-based samples that maintained their structure for 24 h in the presence of the collagenase solution were samples S3 and S5, with a gellan gum content of 25% and 50%, respectively. Regarding sample S6, which also had a 25% gellan gum content, it could be observed that it degraded more quickly, transforming into the corresponding hydrogel, possibly due to the high proportion of HPMC (25%), which, being water-soluble, contributed to the low resistance of this sample.

### 2.8. In Vitro Biocompatibility Assessment

**Figure 12:**
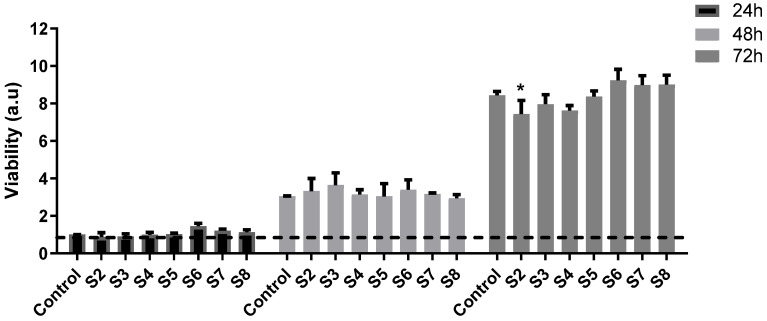
Cell viability determined by XTT assay for human fibroblasts after 24-, 48-, and 72 h incubation with samples’ extracts (* *p* < 0.05 vs. control at 72 h).

At 24 and 48 h post-incubation, the sample extracts showed no cytotoxic effect, as the cell viability was at a similar level to that of the control, as shown in Figure 12. After 3 days, the results showed cell proliferation for all samples; however, the level was lower for S2 with a 7.4-fold increase vs. 8.4 for the control. The highest proliferation was registered for S6, S7, and S8, but without statistical significance compared to the control sample at this time point.

**Figure 13:**
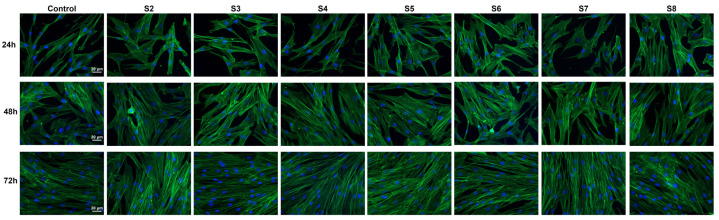
Fluorescence images showing the distribution of F-actin stress fibers (green) for the dermal fibroblasts grown in the same conditions as for the viability testing. Nuclei stained with DAPI are shown in blue.

F-actin immunofluorescence supported the viability results by showing a similar cell pattern as compared to the control. Furthermore, the cells incubated with samples’ extracts maintained their spindle shape with normal stress fiber distribution, as in the case of the controls from each time point, as illustrated in Figure 13.

## 3. Conclusions

This study developed and evaluated biopolymeric formulations based on collagen, HPMC, and GG for potential intravaginal application. The main focus of this study was the physicochemical characterization and biocompatibility evaluation of some biopolymeric structural supports as an important preliminary step in the future development of vaginal drug delivery systems. The rheological analysis confirmed a desirable pseudoplastic, non-Newtonian behaviour that could support effective administration and mucosal spread. The circular dichroism and FT-IR analyses verified the preservation of collagen’s triple-helical structure across the formulations—crucial for maintaining the mechanical and biological properties of collagen—for its use in biomedical applications such as drug delivery or tissue regeneration. The SEM images revealed different morphological characteristics that were aligned with swelling and wettability data. Notably, the gellan gum-based samples demonstrated improved hydrophilicity, structural integrity in simulated vaginal fluid, and greater enzymatic resistance. The mixed samples also achieved a balanced hydration profile, which could contribute to ensuring good contact with the mucosa and increased retention time at the site of action. In terms of cytotoxicity, the S2–S8 samples showed no cytotoxic effects and allowed cell proliferation without inducing F-actin stress fiber modifications. Overall, the developed hydrogels and freeze-dried wafers show promise as platforms for developing vaginal drug delivery systems, with potential biomedical applications in the management of various gynecological conditions.

## 4. Materials and Methods

### 4.1. Materials

Type I collagen gel with a concentration of 2.38% (*w/w*) and acidic pH values was extracted from calf hide by using the technique developed and performed at the Collagen Department of the Division of Leather and Footwear Research Institute, National Research and Development Institute for Textiles and Leather (INCDTP) from Bucharest, Romania. Hydroxypropyl methylcellulose (HPMC) was obtained from Fluka™ (Steinheim, Germany), and the gellan gum was purchased from Thermo Fisher Scientific (Waltham, MA, USA). Sodium hydroxide (NaOH) was from Merck (Darmstadt, Germany), and all other substances used were of analytical grade. The water used both in the preparation process and in the performed analyzes was ultrapure type I water, produced with a Milli-Q EQ 7008 purification system (Merck Millipore).

### 4.2. Methods

#### 4.2.1. Preparation of Individual Hydrogels

Firstly, the three individual hydrogels were prepared. In order to obtain a 1% (*w/v*) collagen gel with physiological pH values (7.2–7.4), 1 M sodium hydroxide (NaOH) solution was added under continuous stirring to the 2.38% collagen gel (acidic pH) extracted from calf hide. The preparation of the 2% (*w/v*) HPMC hydrogel was performed according to the protocol described in our previous study [36]. Briefly, the required amount of polymer was added under continuous mechanical stirring into a Berzelius beaker placed in a water bath. The beaker contained a portion of the ultrapure water intended for preparation, preheated to 80 °C. After obtaining a homogeneous solution, the remaining water, maintained at 4 °C, was gradually added under stirring. The process continued until the polymer was fully hydrated and a transparent gel was obtained. In order to obtain a 1.2% (*w/v*) gellan gum (GG) hydrogel, the required amount of distilled water, heated at 50 °C, was brought into a Berzelius beaker. Then, the appropriate amount of GG was weighed and added by sprinkling it into the beaker under continuous mechanical stirring until a homogeneous gel with a slightly opalescent appearance was obtained. The prepared hydrogels were kept in the refrigerator until use.

#### 4.2.2. Preparation of Freeze-Dried Hydrogels

The combined hydrogels were obtained by mixing the 1% collagen gel (pH = 7.2–7.4) with 2% HPMC and/or 1.2% GG hydrogels, using different percentages, as presented in Table 2. Thus, 8 hydrogels were obtained, of which 3 were control gels (S1—collagen 1%, S7—HPMC 2%, and S8—GG 1.2%) and 5 were combined hydrogels (S2–S6).

**Figure 14:**
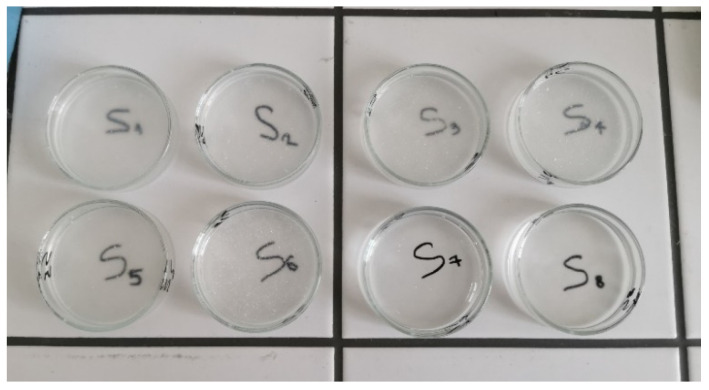
Combined hydrogels placed in Petri dishes (Ø = 5 cm) for lyophilization.

Then, a predetermined amount of the obtained hydrogels was brought into Petri dishes (Figure 14) and freeze-dried using a Delta LSC 2–24 Martin Christ lyophilizer (Osterode am Harz, Germany), following the protocol developed and performed at the Collagen Department of Division Leather and Footwear Research Institute, INCDTP from Bucharest, Romania. Thus, the hydrogels were transferred into glass Petri dishes and placed in a pre-cooled lyophilizer set at −40 °C. The lyophilization process comprised three stages: freezing, primary freeze-drying, and final freeze-drying. The freezing stage was carried out at −40 °C for 8 h. This was followed by the primary freeze-drying phase, conducted at a constant pressure of 0.1 mbar. During this phase, the temperature was gradually increased: from −40 °C to +10 °C over 10 h, then to +20 °C over the next 10 h, and finally to +30 °C over 7 h, with an additional 7 h of maintaining at +30 °C and 0.1 mbar pressure. The final freeze-drying stage was performed at 35 °C and 0.001 mbar for 6 h. After completion of the lyophilization program, the obtained freeze-dried hydrogels (wafers) were carefully detached from the Petri dishes and stored at room temperature in a low-humidity environment until use. The resulting wafers were coded in the same way as the hydrogels (S1–S8).

#### 4.2.3. Evaluation of the Rheological Behavior and Properties of Designed Hydrogels

The evaluation of the rheological behavior and properties of the collagen-, HPMC-, and GG-based hydrogels was performed using a MultiVisc rotational viscometer from Fungilab (Barcelona, Spain), using one of the standard spindles, TR 8. The hydrogels were tested at 23 °C, and the rotational speed range used was 0.3–60 rpm in order to simulate and analyze the rheological behavior of the gels under different shear rates that could be associated with their application and post-application dynamics at the vaginal level.

#### 4.2.4. Circular Dichroism (CD)

The hydrogels that were based on collagen and the two selected biopolymers (S1–S6) were analyzed by circular dichroism (CD) by using the sample S1 (100% collagen) as a control. By means of this analysis method, the potential changes induced by the addition of HPMC and gellan gum gels to the triple-helical structure of collagen were investigated, with the final aim of confirming the maintenance of the structural integrity of collagen within the designed hydrogels and its compatibility with the two selected mucoadhesive biopolymers. The analysis was performed using a Jasco Model J-1500 spectrophotometer (Tokyo, Japan). Briefly, 2 mL of each collagenic hydrogel was introduced into a cylindrical quartz cuvette with an optical path length of 10 mm. Scans were performed in the 250–190 nm spectral region, and spectra were generated using a scan rate of 100 nm/min.

#### 4.2.5. Fourier Transform Infrared (FT-IR) Spectroscopy

Spectral characteristics of the collagen, HPMC, and GG-based freeze-dried hydrogels were obtained using a Fourier Transform Infrared (FT-IR) spectroscopic method, using a JASCO FT-IR 4200 spectrometer (Tokyo, Japan). Data acquisition was performed in the 4000–800 cm^−1^ spectral range, using a resolution of 4 cm^−1^. Each obtained spectrum is the average of 30 scans per sample.

#### 4.2.6. Scanning Electron Microscopy (SEM)

A morphological assessment of the freeze-dried hydrogels was carried out by scanning electron microscopy (SEM), using a TM 4000 Plus apparatus (Hitachi, Tokyo, Japan). The samples were analyzed at 100× magnification, without being coated with a conductive layer, using an accelerating voltage of 15 kV and BSE detection.

#### 4.2.7. Surface Wettability of Freeze-Dried Hydrogels

In order to study the surface wettability of the wafers, the contact angle method was performed by using a KSV Cam 101 Scientific Instrument that was equipped with a digital camera (KSV Instruments, Helsinki, Finland). The study was performed using 37 °C simulated vaginal fluid as the testing liquid. The shape of the drops on the surface of the wafers was monitored by means of the digital camera, and the values of the initial contact angle were recorded. The experiment was performed in triplicate, and the reported values represent the average of three determinations.

#### 4.2.8. Swelling Capacity of Freeze-Dried Hydrogels

The swelling capacity of the freeze-dried hydrogels (wafers) was evaluated in order to study the behavior of the matrices in contact with biological fluids. For this analysis, simulated vaginal fluid (SVF) was used, whose composition was established by Owen and Katz [103]. The study was carried out over a period of 72 h by using the prepared vaginal fluid that was brought to a temperature of 37 °C in order to simulate the conditions found at the site of administration. Initially, approximately equal pieces were cut from the obtained wafers (S1–S8), which were then weighed in their dry form. Each piece was placed in a well, adding 2.5 mL of simulated vaginal fluid. The selected SVF volume reflects the conditions found at the administration site, given the fact that the volume of vaginal fluid secreted in a day is relatively low [94]. At well-established time intervals (0.17; 0.33; 0.5; 1; 1.5; 2; 3; 4; 24; 48; 72 h), each sample was withdrawn from the well, suspended in air for 10 s to remove excess fluid, and then weighed. The experiment was performed in triplicate, and the reported values represent the average of 3 determinations. To evaluate the swelling capacity, the following calculation formula was used (Equation (2)):

Water uptake capacity (g/g) = (W_t_ − W_i_)/W_i_ (2)

where W_t_ represents the weight of the swollen wafer at specific time intervals, and W_i_ is the weight of the wafer in the dry state.

#### 4.2.9. Enzymatic Degradation of Freeze-Dried Hydrogels

In order to investigate the degree of enzymatic degradation suffered by the wafers in the presence of collagenase solution, approximately equal pieces of the spongious matrices were cut and soaked for 24 h in SVF. Later, the SVF was removed, and 2.5 mL of collagenase solution was added over the previously soaked matrices. The samples were kept in the oven at a temperature of 37 °C to simulate physiological temperature. The samples were weighed at predetermined time intervals (0.25; 0.5; 1; 2; 3; 4; 24; 48 and 72 h), and the weight loss was calculated according to Equation (3):

Weight loss (%) = (W_0_ − W_t_)/W_0_ × 100 (3)

where W_0_ represents the initial weight of the wafers soaked in SVF for 24 h, and W_t_ represents the mass of the wafers immersed in collagenase solution at different time points.

The experiment was performed in triplicate, and the reported values represent the average of 3 determinations.

#### 4.2.10. In Vitro Biocompatibility Assessment

In order to evaluate the samples’ cytocompatibility and capacity to sustain cell proliferation, we used human primary dermal fibroblasts from our laboratory cell collection, which were previously isolated and characterized by our group [104]. The cells were seeded at a density of 10^4^/cm^2^ in low glucose DMEM (Sigma Aldrich, St. Louis, MO, USA), supplemented with 10% FBS and 1% antibiotic and maintained at 37 °C, 5% CO_2_ in a humid incubator for routine culture.

Extracts from the samples were prepared by incubating 0.01 g of the material in 5 mL of complete growth medium at 37 °C for 8 h with agitation. The resulted solutions were sterilized using 0.22 µm syringe filters and kept at 4 °C.

Cells were seeded in 96-well plates, and after 24 h, the medium was changed with 100 of % the samples’ extracts. The cell viability was evaluated at three time points: 24-, 48-, and 72-h post-incubation by using the XTT assay (Invitrogen, Carlsbad, CA, USA). Briefly, the cells were washed with warm PBS and incubated with the XTT working solution. After 2 h in the incubator, the absorbance was read at 450 nm vs. 690 nm. The results were expressed as a fold increase of the absorbance reported for the control sample (complete growth medium) at 24 h. Data were represented as the mean ± standard deviation. Statistical significance was tested by one-way ANOVA using GraphPad Prism 9.1, with a *p*-value < 0.05 considered significant.

For the morphological changes that were induced by the samples’ extracts, the cells were seeded on glass cover slips and incubated as described above. At the same time points as for the XTT test, the fibroblasts were fixed with 4% paraformaldehyde and permeabilized with 0.1% Triton X. Phalloidin-Alexa 488 (Thermo Fisher, Waltham, MA, USA) was used as per the manufacturer’s instructions in order to stain the actin filaments. The nuclei were stained with DAPI (Sigma Aldrich, St. Louis, MO, USA), and the cells were examined using a Zeiss Observer D1 microscope.

## Figures and Tables

**Table 1:** Obtained values of m and n parameters and the coefficient of determination R^2^, specific to the Power law model, for hydrogels S1–S8, analyzed at 23 °C.

Sample	S1	S2	S3	S4	S5	S6	S7	S8
m	3.412	3.741	5.212	4.017	6.327	4.583	12.218	10.365
n	0.68	0.62	0.75	0.67	0.71	0.56	0.35	0.69
R^2^	0.9960	0.9990	0.9990	0.9992	0.9985	0.9999	0.9992	0.9987

**Table 2:** Percentage composition of prepared hydrogels and freeze-dried hydrogels (wafers).

Sample	1% Collagen Gel, % *	2% HPMC Gel, % *	1.2% GG Gel, % *
S1	100	0	0
S2	75	25	0
S3	75	0	25
S4	75	12.5	12.5
S5	50	0	50
S6	50	25	25
S7	0	100	0
S8	0	0	100

* Percentages of collagen, HPMC, and GG hydrogels are reported to 100 g gel.

## Data Availability

The data presented in this research are available in the article.

## References

[1] Brako F., Boateng J. (2025). Transmucosal drug delivery: Prospects, challenges, advances, and future directions. Expert Opin. Drug Deliv..

[2] Goyal A.K., Singh R., Chauhan G., Rath G. (2018). Non-invasive systemic drug delivery through mucosal routes. Artif. Cells Nanomed. Biotechnol..

[3] Subi M.T.M., Selvasudha N., Vasanthi H.R. (2024). Vaginal drug delivery system: A promising route of drug administration for local and systemic diseases. Drug Discov. Today.

[4] Pandey M., Choudhury H., Abdul-Aziz A., Bhattamisra S.K., Gorain B., Carine T., Wee Toong T., Yi N.J., Win Yi L. (2020). Promising Drug Delivery Approaches to Treat Microbial Infections in the Vagina: A Recent Update. Polymers.

[5] Ostróżka-Cieślik A., Michalak M., Bryś T., Kudła M. (2025). The Potential of Hydrogel Preparations Containing Plant Materials in Supporting the Treatment of Vaginal and Vulvar Infections—Current State of Knowledge. Polymers.

[6] De Rosa N., Santangelo F., Todisco C., Dequerquis F., Santangelo C. (2023). Collagen-Based Ovule Therapy Reduces Inflammation and Improve Cervical Epithelialization in Patients with Fungal, Viral, and Bacterial Cervico-Vaginitis. Medicina.

[7] Naumova I., Castelo-Branco C. (2018). Current treatment options for postmenopausal vaginal atrophy. Int. J. Womens Health.

[8] Major I., McConville C. (2017). Vaginal drug delivery for the localised treatment of cervical cancer. Drug Deliv. Transl. Res..

[9] Yang Z., Wu X., Wang H., Zhou J., Lin X., Yang P. (2024). Vagina, a promising route for drug delivery. J. Drug Deliv. Sci. Technol..

[10] Osmałek T., Froelich A., Jadach B., Tatarek A., Gadziński P., Falana A., Gralińska K., Ekert M., Puri V., Wrotyńska-Barczyńska J. (2021). Recent Advances in Polymer-Based Vaginal Drug Delivery Systems. Pharmaceutics.

[11] Pires P.C., Mascarenhas-Melo F., Pedrosa K., Lopes D., Lopes J., Macário-Soares A., Peixoto D., Giram P.S., Veiga F., Paiva-Santos A.C. (2023). Polymer-based biomaterials for pharmaceutical and biomedical applications: A focus on topical drug administration. Eur. Polym. J..

[12] Remiro P.F.R., Nagahara M.H.T., Azoubel R.A., Franz-Montan M., d’Ávila M.A., Moraes Â.M. (2022). Polymeric Biomaterials for Topical Drug Delivery in the Oral Cavity: Advances on Devices and Manufacturing Technologies. Pharmaceutics.

[13] Fernandes A.I., Jozala A.F. (2022). Polymers Enhancing Bioavailability in Drug Delivery. Pharmaceutics.

[14] Jawadi Z., Yang C., Haidar Z.S., Santa Maria P.L., Massa S. (2022). Bio-Inspired Muco-Adhesive Polymers for Drug Delivery Applications. Polymers.

[15] Pornpitchanarong C., Rojanarata T., Opanasopit P., Ngawhirunpat T., Bradley M., Patrojanasophon P. (2022). Maleimide-functionalized carboxymethyl cellulose: A novel mucoadhesive polymer for transmucosal drug delivery. Carbohydr. Polym..

[16] Partenhauser A., Bernkop-Schnürch A. (2016). Mucoadhesive polymers in the treatment of dry X syndrome. Drug Discov. Today.

[17] Tong X., Pan W., Su T., Zhang M., Dong W., Qi X. (2020). Recent advances in natural polymer-based drug delivery systems. React. Funct. Polym..

[18] He Q., Feng T., Xie Y., Swamiappan S., Zhou Y., Zhou Y., Zhou H., Peng X. (2025). Recent Advances in the Development and Application of Cell-Loaded Collagen Scaffolds. Int. J. Mol. Sci..

[19] Alberts A., Bratu A.G., Niculescu A.-G., Grumezescu A.M. (2025). Collagen-Based Wound Dressings: Innovations, Mechanisms, and Clinical Applications. Gels.

[20] Tudoroiu E.-E., Albu Kaya M.G., Titorencu I., Dinu-Pîrvu C.E., Marin M.M., Roșca A.-M., Popa L., Anuța V., Antoniac A., Chelaru C. (2023). Design and evaluation of new wound dressings based on collagen-cellulose derivatives. Mater. Des..

[21] Parenteau-Bareil R., Gauvin R., Berthod F. (2010). Collagen-Based Biomaterials for Tissue Engineering Applications. Materials.

[22] Zhou N., Liu Y.D., Zhang Y., Gu T.W., Peng L.H. (2023). Pharmacological Functions, Synthesis, and Delivery Progress for Collagen as Biodrug and Biomaterial. Pharmaceutics.

[23] Ghica M.V., Albu M.G., Popa L., Moisescu S. (2013). Response surface methodology and Taguchi approach to assess the combined effect of formulation factors on minocycline delivery from collagen sponges. Pharmazie.

[24] Tihan G.T., Rău I., Zgârian R.G., Ungureanu C., Barbaresso R.C., Kaya M.G.A., Dinu-Pîrvu C., Ghica M.V. (2019). Oxytetracycline versus Doxycycline Collagen Sponges Designed as Potential Carrier Supports in Biomedical Applications. Pharmaceutics.

[25] Jadach B., Mielcarek Z., Osmałek T. (2024). Use of Collagen in Cosmetic Products. Curr. Issues Mol. Biol..

[26] Photiou L., Lin M.J., Dubin D.P., Lenskaya V., Khorasani H. (2020). Review of non-invasive vulvovaginal rejuvenation. J. Eur. Acad. Dermatol. Venereol..

[27] Wang Y., Wang Z., Dong Y. (2023). Collagen-Based Biomaterials for Tissue Engineering. ACS Biomater. Sci. Eng..

[28] Chen H., Xue L., Gong G., Pan J., Wang X., Zhang Y., Guo J., Qin L. (2022). Collagen-based materials in reproductive medicine and engineered reproductive tissues. J. Leather Sci. Eng..

[29] Gurumurthy B., Janorkar A.V. (2021). Improvements in mechanical properties of collagen-based scaffolds for tissue engineering. Curr. Opin. Biomed. Eng..

[30] Shapiro R.L., DeLong K., Zulfiqar F., Carter D., Better M., Ensign L.M. (2022). In vitro and ex vivo models for evaluating vaginal drug delivery systems. Adv. Drug Deliv. Rev..

[31] Dong C., Lv Y. (2016). Application of Collagen Scaffold in Tissue Engineering: Recent Advances and New Perspectives. Polymers.

[32] Mady O.Y., Dewedar O., Abdine N., Zaytoon H., Haggag Y. (2024). Bioadhesive behaviors of HPMC E5: Comparative analysis of various techniques, histological and human radiological evidence. Sci. Rep..

[33] Hernández-González M.E., Rodríguez-González C.A., Valencia-Gómez L.E., Hernández-Paz J.F., Jiménez-Vega F., Salcedo M., Olivas-Armendáriz I. (2024). Characterization of HPMC and PEG 400 Mucoadhesive Film Loaded with Retinyl Palmitate and Ketorolac for Intravaginal Administration. Int. J. Mol. Sci..

[34] Long Y., Zhao X., Liu S., Chen M., Liu B., Ge J., Jia Y.-G., Ren L. (2018). Collagen–Hydroxypropyl Methylcellulose Membranes for Corneal Regeneration. ACS Omega.

[35] Ding C., Zhang M., Li G. (2015). Preparation and characterization of collagen/hydroxypropyl methylcellulose (HPMC) blend film. Carbohydr. Polym..

[36] Luca I., Albu Kaya M.G., Titorencu I., Dinu-Pîrvu C.E., Marin M.M., Popa L., Rosca A.M., Antoniac A., Anuta V., Prisada R.M. (2025). Influence of Mucoadhesive Polymers on Physicochemical Features and Biocompatibility of Collagen Wafers. ACS Polym. Au.

[37] Vigani B., Rossi S., Sandri G., Bonferoni M.C., Caramella C.M., Ferrari F. (2020). Recent Advances in the Development of In Situ Gelling Drug Delivery Systems for Non-Parenteral Administration Routes. Pharmaceutics.

[38] Abdl Aali R.A., Al-Sahlany S.T. (2024). Gellan Gum as a Unique Microbial Polysaccharide: Its Characteristics, Synthesis, and Current Application Trends. Gels.

[39] Mydin R.B.S.M.N., Alam M., Raman S., Parumasivam T., Shanker K., Mohd Noor S.N.F., Kamaruddin A.F., Navanita S.K., Hadi M.A., Vemireddy B.G.R., Nayak A.K., Hasnain M.S. (2024). Chapter 1—An overview of gellan gum sources, properties, and its targeted applications. Application of Gellan Gum as a Biomedical Polymer.

[40] Milivojevic M., Pajic-Lijakovic I., Bugarski B., Nayak A.K., Hasnain M.S., Hasnain M.S., Nayak A.K. (2019). Chapter 6—Gellan gum in drug delivery applications. Natural Polysaccharides in Drug Delivery and Biomedical Applications.

[41] Lalebeigi F., Alimohamadi A., Afarin S., Aliabadi H.A.M., Mahdavi M., Farahbakhshpour F., Hashemiaval N., Khandani K.K., Eivazzadeh-Keihan R., Maleki A. (2024). Recent advances on biomedical applications of gellan gum: A review. Carbohydr. Polym..

[42] Patel P., Patel P. (2015). Formulation and evaluation of clindamycin HCL in situ gel for vaginal application. Int. J. Pharm. Investig..

[43] Permana A.D., Asri R.M., Amir M.N., Himawan A., Arjuna A., Juniarti N., Utami R.N., Mardikasari S.A. (2023). Development of Thermoresponsive Hydrogels with Mucoadhesion Properties Loaded with Metronidazole Gel-Flakes for Improved Bacterial Vaginosis Treatment. Pharmaceutics.

[44] Jalil A., Asim M.H., Le N.N., Laffleur F., Matuszczak B., Tribus M., Bernkop-Schnürch A. (2019). S-protected gellan gum: Decisive approach towards mucoadhesive antimicrobial vaginal films. Int. J. Biol. Macromol..

[45] Pahwa R., Ahuja M. (2023). Nanocellulose-gellan cross-linked scaffolds for vaginal delivery of fluconazole. Int. J. Biol. Macromol..

[46] Biswal S., Parmanik A., Das D., Sahoo R.N., Nayak A.K. (2025). Gellan gum-based in-situ gel formulations for ocular drug delivery: A practical approach. Int. J. Biol. Macromol..

[47] Pahwa R., Sharma R., Ahuja M., Nayak A.K., Hasnain M.S. (2024). Chapter 22—Gellan gum–based ocular formulations. Application of Gellan Gum as a Biomedical Polymer.

[48] Jelkmann M., Leichner C., Zaichik S., Laffleur F., Bernkop-Schnürch A. (2020). A gellan gum derivative as in-situ gelling cationic polymer for nasal drug delivery. Int. J. Biol. Macromol..

[49] Sipos B., Budai-Szűcs M., Katona G., Csóka I. (2025). Gellan Gum-Based In Situ Hydrogels for Nasal Delivery of Polymeric Micelles Loaded with Risperidone. Gels.

[50] Li A., Khan I.N., Khan I.U., Yousaf A.M., Shahzad Y. (2021). Gellan Gum-Based Bilayer Mucoadhesive Films Loaded with Moxifloxacin Hydrochloride and Clove Oil for Possible Treatment of Periodontitis. Drug Des. Dev. Ther..

[51] Prezotti F.G., Siedle I., Boni F.I., Chorilli M., Müller I., Cury B.S.F. (2020). Mucoadhesive films based on gellan gum/pectin blends as potential platform for buccal drug delivery. Pharm. Dev. Technol..

[52] Osmari B.F., Giuliani L.M., Reolon J.B., Rigo G.V., Tasca T., Cruz L. (2020). Gellan gum-based hydrogel containing nanocapsules for vaginal indole-3-carbinol delivery in trichomoniasis treatment. Eur. J. Pharm. Sci..

[53] Wu S., Xiao R., Wu Y., Xu L. (2024). Advances in tissue engineering of gellan gum-based hydrogels. Carbohydr. Polym..

[54] Dodi G., Sabau R.E., Crețu B.E.B., Gardikiotis I. (2023). Exploring the Antioxidant Potential of Gellan and Guar Gums in Wound Healing. Pharmaceutics.

[55] Gong Y., Wang C., Lai R.C., Su K., Zhang F., Wang D.-a. (2009). An improved injectable polysaccharide hydrogel: Modified gellan gum for long-term cartilage regenerationin vitro. J. Mater. Chem..

[56] Oliveira J.T., Gardel L.S., Rada T., Martins L., Gomes M.E., Reis R.L. (2010). Injectable gellan gum hydrogels with autologous cells for the treatment of rabbit articular cartilage defects. J. Orthop. Res..

[57] Silva-Correia J., Oliveira J.M., Caridade S.G., Oliveira J.T., Sousa R.A., Mano J.F., Reis R.L. (2011). Gellan gum-based hydrogels for intervertebral disc tissue-engineering applications. J. Tissue Eng. Regen. Med..

[58] Pereira D.R., Silva-Correia J., Oliveira J.M., Reis R.L., Pandit A., Biggs M.J. (2018). Nanocellulose reinforced gellan-gum hydrogels as potential biological substitutes for annulus fibrosus tissue regeneration. Nanomed. Nanotechnol. Biol. Med..

[59] Alheib O., da Silva L.P., da Silva Morais A., Mesquita K.A., Pirraco R.P., Reis R.L., Correlo V.M. (2022). Injectable laminin-biofunctionalized gellan gum hydrogels loaded with myoblasts for skeletal muscle regeneration. Acta Biomater..

[60] Liu H., Li K., Guo B., Yuan Y., Ruan Z., Long H., Zhu J., Zhu Y., Chen C. (2024). Engineering an injectable gellan gum-based hydrogel with osteogenesis and angiogenesis for bone regeneration. Tissue Cell.

[61] Feketshane Z., Alven S., Aderibigbe B.A. (2022). Gellan Gum in Wound Dressing Scaffolds. Polymers.

[62] Mahmod Z., Zulkifli M.F., Masimen M.A.A., Ismail W.I.W., Sharifudin M.A., Amin K.A.M. (2025). Investigating the efficacy of gellan gum hydrogel films infused with Acacia stingless bee honey in wound healing. Int. J. Biol. Macromol..

[63] Shaker D.S., Ismail S., Hamed S., El-Shishtawy E.M. (2018). Butoconazole nitrate vaginal sponge: Drug release and antifungal efficacy. J. Drug Deliv. Sci. Technol..

[64] Furst T., Piette M., Lechanteur A., Evrard B., Piel G. (2015). Mucoadhesive cellulosic derivative sponges as drug delivery system for vaginal application. Eur. J. Pharm. Biopharm..

[65] Hashempur M.H., Radmanesh A., Karami F., Zomorodian K., Amirzadeh N., Shenavari S., Zareshahrabadi Z. (2025). Fabrication of a sponge-like protein based hydrogel incorporating fluconazole against Candida species as a potential treatment for vulvovaginal candidiasis infection. Sci. Rep..

[66] Brandão Reolon J., Hammerschmitt B., Sari M.H., Luna Lazo R.E., de Fátima A., Capeletti M., Rigo M., Bonini J.S., Abaide A., Pontarolo R. (2024). Predictive Modeling of Rheological Behavior in Semisolid Pharmaceutical Formulations Using Computational Tools. Braz. Arch. Biol. Technol..

[67] das Neves J., da Silva M.V., Gonçalves M.P., Amaral M.H., Bahia M.F. (2009). Rheological properties of vaginal hydrophilic polymer gels. Curr. Drug Deliv..

[68] Ghica M.V., Hîrjău M., Lupuleasa D., Dinu-Pîrvu C.-E. (2016). Flow and Thixotropic Parameters for Rheological Characterization of Hydrogels. Molecules.

[69] Herrada-Manchón H., Fernández M.A., Aguilar E. (2023). Essential Guide to Hydrogel Rheology in Extrusion 3D Printing: How to Measure It and Why It Matters?. Gels.

[70] Yu T., Malcolm K., Woolfson D., Jones D.S., Andrews G.P. (2011). Vaginal gel drug delivery systems: Understanding rheological characteristics and performance. Expert Opin. Drug Deliv..

[71] Wang K., Cao R., Dong H. (2025). Diversity of Collagen Proteins and Their Biomedical Applications in Drug Delivery. Appl. Sci..

[72] Kirkness M.W., Lehmann K., Forde N.R. (2019). Mechanics and structural stability of the collagen triple helix. Curr. Opin. Chem. Biol..

[73] Wosicka-Frąckowiak H., Poniedziałek K., Woźny S., Kuprianowicz M., Nyga M., Jadach B., Milanowski B. (2024). Collagen and Its Derivatives Serving Biomedical Purposes: A Review. Polymers.

[74] Fields G.B. (1995). The collagen triple-helix: Correlation of conformation with biological activities. Connect. Tissue Res..

[75] Malcor J.D., Mallein-Gerin F. (2022). Biomaterial functionalization with triple-helical peptides for tissue engineering. Acta Biomater..

[76] Marin M.M., Kaya M., Vlăsceanu G., Ghitman J., Radu I.-C., Iovu H. (2021). The Effect of Crosslinking Agents on the Properties of Type II Collagen Biomaterials. Mater. Plast..

[77] Tronci G., Russell S.J., Wood D.J. (2013). Photo-active collagen systems with controlled triple helix architecture. J. Mater. Chem. B.

[78] Fathima N., Devi R., Rekha K., Dhathathreyan A. (2009). Collagen-curcumin interaction—A physico-chemical study. J. Chem. Sci..

[79] Gao J., Ning C., Wang M., Wei M., Ren Y., Li W. (2024). Structural, antioxidant activity, and stability studies of jellyfish collagen peptide–calcium chelates. Food Chem. X.

[80] Zhang X., Xu S., Shen L., Li G. (2020). Factors affecting thermal stability of collagen from the aspects of extraction, processing and modification. J. Leather Sci. Eng..

[81] Riaz T., Zeeshan R., Zarif F., Ilyas K., Muhammad N., Safi S., Rahim A., Rizvi S.A.A., Rehman I.U. (2018). FTIR analysis of natural and synthetic collagen FTIR analysis of natural and synthetic collagen. Appl. Spectrosc. Rev..

[82] Jiang H., Kong Y., Song L., Liu J., Wang Z. (2023). A Thermostable Type I Collagen from Swim Bladder of Silver Carp (Hypophthalmichthys molitrix). Mar. Drugs.

[83] Furqan M., Iqbal F., Tulain R. (2017). Microwave radiation induced synthesis of hydroxypropyl methylcellulose-graft-(polyvinylalcohal-co-acrylic acid) polymeric network and its in vitro evaluation. Acta Pol. Pharm.-Drug Res..

[84] Verma A., Dixit R., Singh U., Soni S., Mishra A., Bansal A., Pandit J. (2011). Preparation and Characterization of Gellan-Chitosan Polyelectrolyte Complex Beads. Lat. Am. J. Pharm..

[85] Shakeri M.-s., Naji-Tabasi S. (2024). Characterization and optimization of gellan gum production by natural Sphingomonas sp. SM2. LWT.

[86] Vranceanu M., Şaban R., Antoniac I., Albu M., Miculescu F. (2012). Development and characterization of novel porous collagen based biocomposite for bone tissue regeneration. UPB Sci. Bull. Ser. B Chem. Mater. Sci..

[87] Szychlinska M.A., Calabrese G., Ravalli S., Dolcimascolo A., Castrogiovanni P., Fabbi C., Puglisi C., Lauretta G., Di Rosa M., Castorina A. (2020). Evaluation of a Cell-Free Collagen Type I-Based Scaffold for Articular Cartilage Regeneration in an Orthotopic Rat Model. Materials.

[88] Naji-Tabasi S., Shahidi-Noghabi M., Dovom A. (2023). Investigating the fabrication and functional properties of new composite hydrogels containing gellan/alginate/xanthan gum. J. Sol-Gel Sci. Technol..

[89] Rupenthal I., Green C., Alany R. (2011). Comparison of ion-activated in situ gelling systems for ocular drug delivery. Part 1: Physicochemical characterisation and in vitro release. Int. J. Pharm..

[90] Rojewska M., Olejniczak-Rabinek M., Bartkowiak A., Snela A., Prochaska K., Lulek J. (2017). The wettability and swelling of selected mucoadhesive polymers in simulated saliva and vaginal fluids. Colloids Surf. B Biointerfaces.

[91] Popa L., Ghica M.V., Albu M.G., Ortan A., Dinu-Pîrvu C.E. (2013). Hysteresis of contact angle. Dynamic wettability studies of collagen and doxycycline porous matrices crosslinked with tannic acid. Dig. J. Nanomater. Biostruct..

[92] Rohan L.C., Sassi A.B. (2009). Vaginal drug delivery systems for HIV prevention. AAPS J..

[93] Adnane M., Meade K.G., O’Farrelly C. (2018). Cervico-vaginal mucus (CVM)—An accessible source of immunologically informative biomolecules. Vet. Res. Commun..

[94] Tietz K., Klein S. (2018). Simulated Genital Tract Fluids and Their Applicability in Drug Release/Dissolution Testing of Vaginal Dosage Forms. Dissolut. Technol..

[95] Carbinatto F.M., de Castro A.D., Evangelista R.C., Cury B.S.F. (2014). Insights into the swelling process and drug release mechanisms from cross-linked pectin/high amylose starch matrices. Asian J. Pharm. Sci..

[96] Deshmukh K., Basheer Ahamed M., Deshmukh R.R., Khadheer Pasha S.K., Bhagat P.R., Chidambaram K., Sadasivuni K.K., Ponnamma D., Kim J., Cabibihan J.J., AlMaadeed M.A. (2017). 3-Biopolymer Composites With High Dielectric Performance: Interface Engineering. Biopolymer Composites in Electronics.

[97] Muthukumar T., Song J.E., Khang G. (2019). Biological Role of Gellan Gum in Improving Scaffold Drug Delivery, Cell Adhesion Properties for Tissue Engineering Applications. Molecules.

[98] Szymańska E., Wojasiński M., Dąbrowska J., Krzyżowska M., Nowicka M., Ciach T., Winnicka K. (2022). Chitosan-poly(ethylene oxide) nanofibrous mat as a vaginal platform for tenofovir disoproxyl fumarate—The effect of vaginal pH on drug carrier performance. Int. J. Biol. Macromol..

[99] Teodora Tihan G., Ungureanu C., Constantin Barbaresso R., Gabriela Zgârian R., Rău I., Meghea A., Georgiana Albu M., Violeta Ghica M. (2015). Chloramphenicol collagen sponges for local drug delivery in dentistry. Comptes Rendus Chim..

[100] Onderdonk A.B., Delaney M.L., Fichorova R.N. (2016). The Human Microbiome during Bacterial Vaginosis. Clin. Microbiol. Rev..

[101] Łaniewski P., Herbst-Kralovetz M.M. (2021). Bacterial vaginosis and health-associated bacteria modulate the immunometabolic landscape in 3D model of human cervix. NPJ Biofilms Microbiomes.

[102] Lithgow K.V., Buchholz V.C.H., Ku E., Konschuh S., D’Aubeterre A., Sycuro L.K. (2022). Protease activities of vaginal Porphyromonas species disrupt coagulation and extracellular matrix in the cervicovaginal niche. npj Biofilms Microbiomes.

[103] Owen D.H., Katz D.F. (1999). A vaginal fluid simulant. Contraception.

[104] Ţuţuianu R., Roşca A.M., Florea G., Prună V., Iacomi D.M., Rădulescu L.A., Neagu T.P., Lascăr I., Titorencu I.D. (2019). Heterogeneity of human fibroblasts isolated from hypertrophic scar. Rom. J. Morphol. Embryol..

